# Winifred P. Brinckman

**Published:** 1970-04

**Authors:** 


### Winifred P. BRINCKMAN, M.B., Ch.B.

Dr. Winifred P. Brinckman, a former general medical practitioner at Bristol, died on 29 April at the age of 74.

Winifred Peris Brinckman was born on 26 February 1895 at Llanberis, in Caernarvonshire. She was educated at Downe House School, in Kent, and began her professional training at the Royal Free Hospital in London, but her studies were interrupted first by illness and then by the first world war, during which she worked in an aircraft factory. She resumed her medical studies after the war, and graduated M.B., Ch.B. at Bristol University in 1927, taking the Conjoint diploma in the same year. A series of house posts completed her training, and she then started in practice.

She was an outstanding general practitioner, her special interests being diabetes and maternity work, and she made several contributions to the literature on diabetes. She built up a busy and most successful practice, and gave dedicated service to her patients, by whom she will be much missed. About 18 months ago she sustained serious injuries in a road traffic accident, and the disability resulting from this caused her to give up active medical work. She had always been a regular attender at professional meetings and postgraduate courses, working diligently to keep up with modern progress and changes. She was a founder member of the College of General Practitioners, and recently had been made an honorary life member. It was a great disappointment to her to have to retire, but those who knew her were filled with admiration at the courage and patience with which she accepted her misfortunes, and, although in constant pain, her fortitude and cheerfulness were an example to everyone. After some months of retirement she developed an illness which she knew was to be fatal. She met this fact calmly, and set about putting all her affairs in order. She was a regular worshipper at Christ Church, Clifton. Religion was a powerful influence in her life, and her Christian principles and beliefs were a wonderful source of strength and consolation to her in her final illness.

She is deeply mourned by all who had the privilege of knowing her.—G.M.F.

(The above notices appeared in the British Medical Journal on 7th June and 18th October 1969 and are reproduced by permission of the Editor, British Medical Journal and of the authors.)

